# Three new species of the genus *Ripipteryx* from Colombia (Orthoptera, Ripipterygidae)

**DOI:** 10.3897/zookeys.502.8871

**Published:** 2015-05-05

**Authors:** Nathalie Baena-Bejarano, Sam W. Heads

**Affiliations:** 1 Illinois Natural History Survey, University of Illinois at Urbana-Champaign, 1816 South Oak Street, Champaign, Illinois 61820-6960, USA; 2 Department of Entomology, University of Illinois at Urbana-Champaign, Morrill Hall, 505 South Goodwin Avenue, Urbana, Illinois 61801-3707, USA; 3 Instituto de Ciencias Naturales, Universidad Nacional de Colombia, Bogotá, Colombia

**Keywords:** Caelifera, Tridactyloidea, species groups, Neotropics, Colombian National Natural Park

## Abstract

Three new species of *Ripipteryx* Newman (Orthoptera: Tridactyloidea: Ripipterygidae) are described from Colombia; namely *Ripipteryx diegoi* **sp. n.** (Forceps Group) and *Ripipteryx guacharoensis* **sp. n.** (Marginipennis Group) from Parque Nacional Natural Cueva de los Guacharos in Huila, and *Ripipteryx gorgonaensis* **sp. n.** (Crassicornis Group) from Parque Nacional Natural Gorgona in Cauca. *Ripipteryx diegoi* **sp. n.** is characterized by the antennae black with white spots on flagellomeres 3–7, male subgenital plate with median ridge forming a bilobed setose process, epiproct produced laterally near its base and phallic complex with virga thickened distally and not reaching beyond the membrane. *Ripipteryx guacharoensis* **sp. n.** is characterized by the antennae thick with white spots present dorsally on flagellomeres 1–4 and 8, epiproct narrow and triangular, uncus reduced and lacking a distal hook, phallic complex with a concave ventral plate and a dorsal elevation in the middle extended to the virga, and the virga itself with two small projections basally. *Ripipteryx gorgonaensis* **sp. n.** is characterized by the epiproct with a lateral notch, antennae with a white dorsal spot on flagellomere 1 and flagellomeres 4–7 entirely white. The antennal color pattern of *Ripipteryx gorgonaensis* **sp. n.** strongly resembles that of *Ripipteryx atra* but differs from the latter in the absence of any significant morphological modification of the flagellomeres.

## Abstract (es)

Se describen tres nuevas especies de *Ripipteryx* Newman (Orthoptera: Tridactyloidea: Ripipterygidae) provenientes de Colombia; son nombradas *Ripipteryx diegoi* **sp. n.** (Grupo Forceps) y *Ripipteryx guacharoensis* **sp. n.** (Grupo Marginipennis) encontradas en el Parque Nacional Natural Cueva de los Guacharos en Huila y *Ripipteryx gorgonaensis* **sp. n.** encontrada en el Parque Nacional Natural Gorgona en Cauca. *Ripipteryx diegoi* **sp. n.** se reconoce por presentar antenas negras con mancha blanca en flagelómeros 3–7, placa subgenital con proceso medial bilobulado con setas, epiprocto extendido lateralmente cerca a la base y complejo fálico con virga engrosada distalmente que no se extiende más allá de la membrana. *Ripipteryx guacharoensis* **sp. n.** se reconoce por presentar antenas gruesas con mancha dorsal blanca en flagelómeros 1–4 y 8, epiprocto angosto y triangular, uncus reducido y sin gancho distal, y complejo fálico con placa ventral cóncava y con elevación dorsomedial extendida hasta la virga, virga basalmente con dos puntas cortas. *Ripipteryx gorgonaensis* **sp. n.** se caracteriza por presentar epiprocto con muesca lateral, antenas con mancha dorsal blanca en flagelómero 1 y flagelómeros 4–7 completamente blancos. El patrón de coloración antenal en *Ripipteryx gorgonaensis* **sp. n.** es similar al de *Ripipteryx atra* pero se diferencia de esta especie por la ausencia de modificaciones morfológicas en los flagelómeros.

## Introduction

*Ripipteryx* Newman, 1834, or mud crickets (Orthoptera: Tridactyloidea: Ripipterygidae), comprises some 45 species of small, dark-colored, cricket-like orthopterans usually found near rivers, in bare soil, and in the moist zones of gallery forests. Like many of their relatives in the larger cosmopolitan family Tridactylidae, the mud crickets are able to jump from the surface of water. The genus is readily distinguished from *Mirhipipteryx*, the only other genus in the family, by its comparatively larger size (body 5.5–14.0 mm long), interocular distance at least half the width of the compound eyes, metatarsus approximately equal in length to the metatibial spurs, and the distinctly sclerotized lateral valvulae of the phallus (Günther 1969; Heads 2010). Species of the genus are usually black or very dark brown, often with contrasting white, yellow and occasionally red markings (Heads 2010). Some species are a dark metallic blue in life, though this coloration often fades to brown or black after death. While ripipterygids are common in many habitats throughout the Neotropics, they are often overlooked by collectors due both to their small size and their fast and very active movements making it difficult to secure specimens. In addition to the paucity of specimens in collections, chronic under-sampling and the difficulty in studying these insects in the field, means that very little is known of their distribution and basic biology (Heads and Taylor 2012; Baena-Bejarano 2015).

The genus is distributed from Mexico to Argentina with their highest diversity found in Ecuador. Some species are very widely distributed (e.g. *Ripipteryx brunneri* Chopard, 1920, *Ripipteryx carbonaria* Saussure, 1896, *Ripipteryx hydrodroma* Saussure, 1896, *Ripipteryx rivularia* Saussure, 1896, etc.) and are found across large ranges in South and Central America (Günther 1969, 1976, 1980, 1989, 1994), while others are more restricted in distribution. A number of endemic species are known from Peru (*Ripipteryx difformipes* Chopard, 1956, *Ripipteryx furcata* Günther, 1976, *Ripipteryx luteicornis* Chopard, 1920 and *Ripipteryx vicina* Chopard, 1956), Ecuador (*Ripipteryx paraprocessata* Günther, 1989, *Ripipteryx pasochoensis* Heads, 2010 and *Ripipteryx trimaculata* Günther, 1969), Brazil (*Ripipteryx brasiliensis* Günther, 1969, *Ripipteryx lawrencei* Günther, 1969 and *Ripipteryx saopauliensis* Günther, 1969) and Colombia (*Rhipipteryx capotensis* Günther, 1970 and *Ripipteryx sturmi* Günther, 1963) (Günther 1962, 1963, 1969, 1970, 1976, 1989, 1994; Heads 2010). Here, three new species from Colombia are described; namely *Ripipteryx diegoi* sp. n. and *Ripipteryx guacharoensis* sp. n. from Parque Nacional Natural Cueva de los Guacharos in Huila, and *Ripipteryx gorgonaensis* sp. n. from Parque Nacional Natural Gorgona in Cauca.

## Material and methods

**Figure 1.**
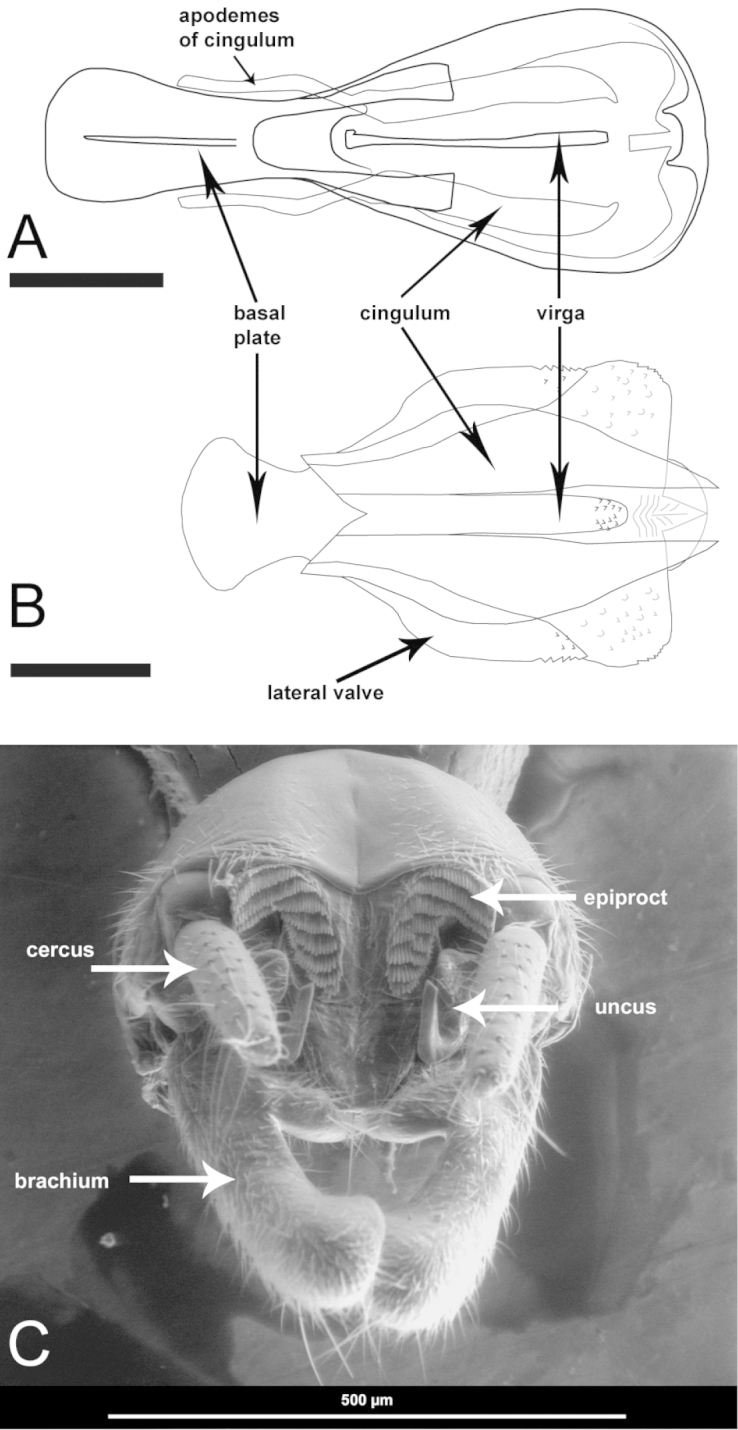
Genitalia and terminalia with labels indicating main morphological features. **A–B** Comparison of phallic complex in dorsal view **A** *Ripipteryx diegoi* sp. n. (IAvH-E 142877) (Scale bar 0.3 mm) **B** *Ripipteryx guacharoensis* sp. n. (IAvH-E 137238) (Scale bar 0.2 mm) **C** *Ripipteryx diegoi* sp. n. scanning electron micrographs of dissected paratype terminalia (IAvH-E 137238) in dorsal view.

The material studied here is deposited in the Instituto de Investigaciones Alexander von Humboldt, Villa de Leyva (IAvH-E) and the entomological museum of Universidad del Valle, Cali (MUSENUV). The male terminalia and the phallic complex were dissected and stored under glycerin in microvials mounted on the pin beneath the specimen. Some specimens were kept in alcohol. The description of morphological characters follows Heads (2010) and Heads and Taylor (2012) (Fig. 1). Interocular distance was measured using a calibrated micrometer slide adapted to a stereomicroscope. Other measurements were made from photographs analyzed with a calibrated digital scale in the program tpsdig2 (Rohlf 2006). Morphological characters were defined and documented in a Delta matrix and the description developed using Delta software (Dallwitz 1980, 1999). Photographs were taken with a Leica digital camera attached to a stereomicroscope and focus-stacked in CombineZP (Hadley 2009). Scanning electron micrographs were produced using FEI Quanta 200 scanning electron microscope. Drawings were produced in Adobe Illustrator CS5 and Photoshop CS5.

## Systematics

### Genus *Ripipteryx* Newman, 1834

#### Forceps Group *sensu* Heads, 2010

##### Ripipteryx diegoi

Taxon classificationAnimaliaOrthopteraRipipterygidae

Baena-Bejarano sp. n.

http://zoobank.org/9AA81DE2-96FB-463B-9B6A-7123BCCB4972

Fig. 2

###### Holotype.

♂ (no. IAvH-E 142877), Colombia, Huila, PNN Cueva de Los Guácharos, Cabaña Cedros, 1°37'N, 76°6'W, 2100 m, Malaise, 6-27.iv.2002, Col. J. Fonseca. Specimen dried and pinned; deposited at Instituto Alexander von Humboldt, Villa de Leyva.

###### Paratypes.

Five specimens from same locality as holotype: 1) ♂ (no. IAvH-E 137238), specimen preserved in alcohol; 2) ♂ (no. IAvH-E 137239), specimen preserved in alcohol; 3) ♀ (no. IAvH-E 137240), specimen preserved in alcohol; 4) ♀ (no. IAvH-E 137241), specimen preserved in alcohol; 5) ♀ (no. IAvH-E 142878), specimen dried and pinned. Specimens deposited at same institution as holotype.

###### Diagnosis.

The new species is almost cryptically similar to *Ripipteryx forceps* Saussure, 1896 in that the uncus is elongate and strongly recurved, and that the median ridge of the male subgenital plate is produced distally, forming a short and densely setose bilobed process. However, it can be readily distinguished from the latter species by [1] antennae with white spots on flagellomeres 3–4 and 6–7 with flagellomere 5 entirely white; [2] epiproct produced laterally near its base; [3] brachium curved along its entire length without prominent apical bulge; and [4] phallic complex with virga thickened distally and not reaching beyond the membrane.

###### Description.

**Figure 2.**
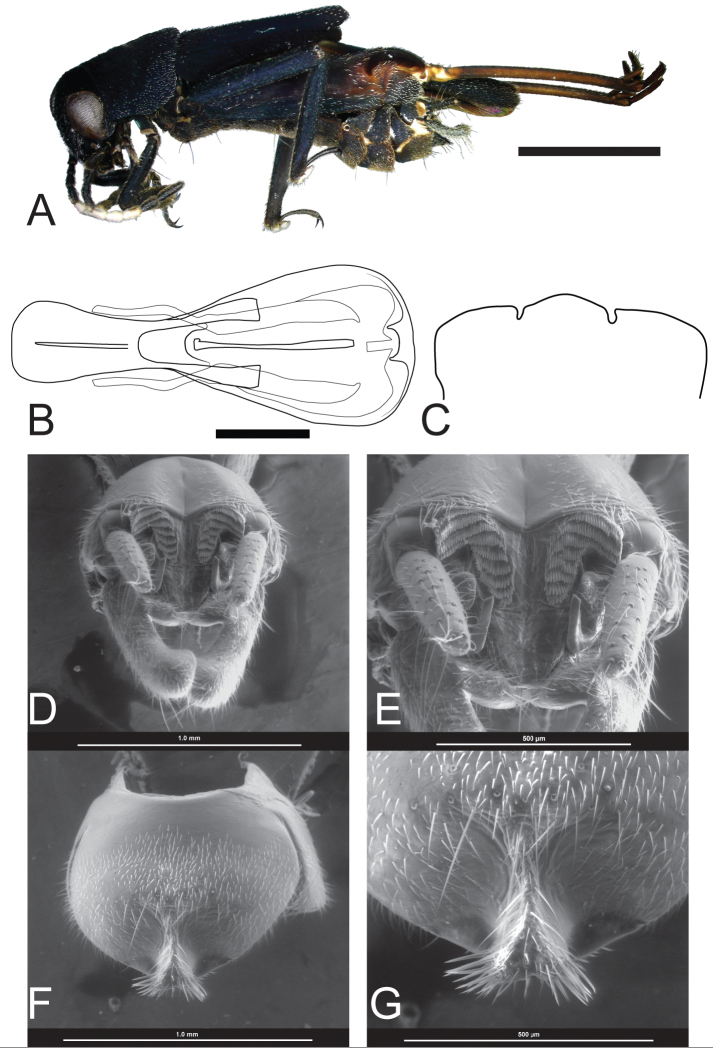
*Ripipteryx diegoi* sp. n. **A** Holotype lateral habitus (IAvH-E 142877) (Scale bar 2.3 mm) **B** Dorsal view of holotype phallic complex (IAvH-E 142877) (Scale bar 0.3 mm) **C** Outline of the subgenital plate in females (ventral view) **D–G** scanning electron micrographs of dissected paratype terminalia (IAvH-E 137238) **D** Dorsal view of terminalia **E** Dorsal view of epiproct and uncus **F** Ventral view of subgenital plate **G** Ventral view of medial bifurcated ridge of subgenital plate.

*Male* (holotype). Body length including wings 8.1 mm, excluding wings 7.9 mm; pronotum length 1.6 mm, pronotum width 1.9 mm; tegmina length 3.1 mm; hind wings length 6.0 mm; interocular distance 0.39 mm. (n=1) (Fig. 2A).

Head. Interocular distance more than half the eye width. Median ocellus fully developed. Patch that circumscribes anterodorsal margin of compound eyes absent. Internal margin of compound eyes convergent dorsally. Maxillary palp black, five segmented, with second segment reduced. Labial palp black. Gena below the compound eye and antennae insertion black.

Antennae black and filiform. Number of antennae segments 10. Scape wider than pedicel. Pedicel as long as 1^st^flagellomere. White spot on scape absent. White spot on pedicel absent. White dorsal spot on flagellomere 1 and 2 absent. White dorsodistal spot on flagellomere 3 present. Flagellomere 4 white with a brownish slender anterior ring. Flagellomere 5 completely white. Flagellomere 6 white with a brownish slender distal ring extended ventrally to the segment half. Flagellomere 7 and 8 black.

Thorax. Pronotum black. Mesonotum black. Tegmina black. Hind wings with white, transverse groove. Procoxa black. Profemora black with an inner distal white spot. Protibiae black with three distal spines and an anterior external white rounded spot close to tibiae-femora articulation. Mesocoxa black. Mesotrochanter black. Mesofemora black. Mesotibiae black. Metafemora black. Semi-lunar process brown. Metatibia brown. Metatarsi brown and longer than metatibial posterior spurs.

Abdomen. Cerci unsegmented, black. Brachium black with a yellow-white distal spot, in lateral view curved along its entire length without prominent apical bulge. Brachium spine present. Epiproct produced laterally near its base. Epiproct lateral lobes narrow. Medial epiproct (distal section) tongue-like. Uncus not embedded in brachium lobe basis, 1-hooked (Fig. 2D, E). Male subgenital plate with a medial bifurcated ridge covered with setae at rounded end (Fig. 2F, G).

Basal plate heavily sclerotized, long, basally strongly widened and distally strongly split. Cingulum with apodemes elongate and well-sclerotized. Sclerotized region of cingulum discontinuous with a distal membranous region in-between. Virga very slender near base and distally thickened. Virga not extended beyond cingulum (Fig. 2B).

*Variations*. Body length including wings 7.9–8.3 mm, excluding wings 7.6–8.3 mm; pronotum length 1.6–1.7 mm, pronotum width 1.9–2.0 mm; tegmina length 3.1–3.2 mm; hind wings length 5.8–6.0 mm; interocular distance 0.39–0.44 mm. (n=3). Antennae: flagellomere 4 white with a brownish slender anterior ring extended ventrally to the segment half. Flagellomere 6 white with a brownish slender distal ring extended ventrally over the segment. Flagellomere 7 white dorsal spot on base.

*Female*. Body similar to male, except for antennal sexual dimorphism and abdominal sexual structures. White dorsodistal spot often present on flagellomere 2. Flagellomere 4 to 7 completely white. Subgenital plate smooth with two distal notches forming a rounded lobe in middle (Fig. 2C). The color is a lighter brown close to the notches.

*Females variation*. body length including wings 7.9–8.6 mm, excluding wings 7.2–8.6 mm; pronotum length 1.7–1.8 mm, pronotum width 2.1–2.2 mm; tegmina length 3.4–4.1 mm; hind wings length 5.5–6.1 mm; interocular distance 0.46–0.47 mm. (n=3). Antenna: White dorsal spot on flagellomere 3 sometimes begins from middle. Flagellomere 7 sometimes presents ventral black color.

###### Etymology.

The specific epithet is patronymic and honours Señor Diego Baena, father of the senior author, in thanks for his care and dedication.

###### Distribution.

This species is currently known from the type locality.

###### Sympatric species.

The new species was found in one of the malaise samples together with *Ripipteryx guacharoensis* and *Ripipteryx ecuadoriensis*, with which it is believed to live sympatrically.

###### Remarks.

*Ripipteryx diegoi* sp. n. is assigned to the Forceps group based on the predominately black coloration, the form of the subgenital plate, morphology of the phallic complex and the body size 7.2-8.7 mm. This species is similar to *Ripipteryx forceps* with which it shares the form of the subgenital plate presenting a median ridge forming a bilobed setose process in ventral view (Fig. 2G). This character allows differentiating it from the other species of the group. Moreover, it differs from *Ripipteryx forceps* by the shape of the terminalia (Fig. 2D) where the epiproct is produced laterally near its base (Fig. 2E), the brachium in lateral view distally curved without prominent apical bulge; (see Günther 1969). Also, the new species differs in the color pattern of the male antennae with white spots on flagellomeres 3 to 7 of *Ripipteryx diegoi* while these are present on the flagelomeres 1, 2, 4 and 5 of *Ripipteryx forceps*.

#### Marginipennis Group *sensu* Heads, 2010

##### Ripipteryx guacharoensis

Taxon classificationAnimaliaOrthopteraRipipterygidae

Baena-Bejarano & Heads sp. n.

http://zoobank.org/DC599EBF-E57F-4266-94C4-3E849483EB6D

Fig. 3

###### Holotype.

♂ (no. IAvH-E 113834), Colombia, Huila, PNN Cueva de Los Guácharos, Cabaña Cedros, 1°37'N, 76°6'W, 2100 m, Malaise 2, 28.xi–2.xii.2001, Col. D. Campos. Specimen dried and pinned; deposited at Instituto Alexander von Humboldt, Villa de Leyva.

###### Paratypes.

Two specimens from same locality as holotype: 1) ♂ (no IAvH-E 137236), 04–18.ii.2001, Col. D. Cortés, specimen preserved in alcohol; 2) ♀ (no. IAvH-E 137237), 27.iv–5.v.2002, Col. J. Fonseca, specimen preserved in alcohol. Specimens deposited at same institution as holotype.

###### Diagnosis.

**Figure 3.**
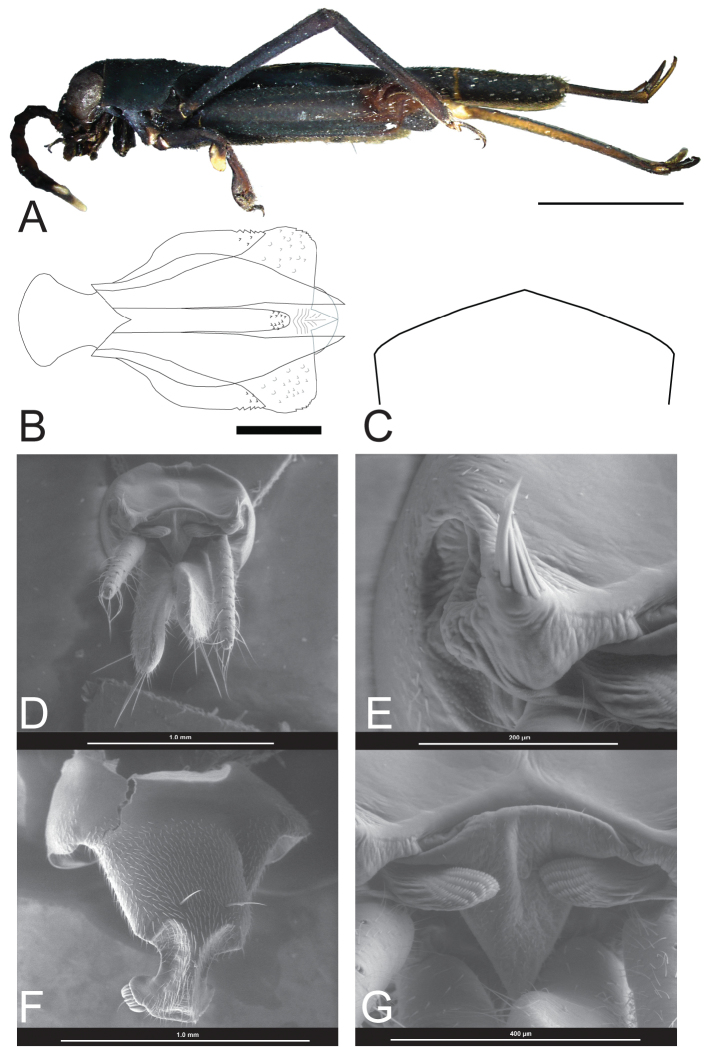
*Ripipteryx guacharoensis* sp. n. **A** Holotype lateral habitus (IAvH-E 113834) (Scale bar 2.2 mm) **B** Dorsal view of phallic complex (IAvH-E 113834) (Scale bar 0.2 mm) **C** Outline of the subgenital plate in females (ventral view) **D–G** scanning electron microscope micrographs of dissected male **D** Dorsal view of terminalia **E** Dorsal view of erect vertical setae at edges **F** Ventral view of subgenital plate **G** Dorsal view of epiproct.

The new species is distinguished from congeners by the following characters: [1] antennae with white dorsal spots on flagellomeres 1–4 and flagellomere 8 black with the distal half completely white; [2] epiproct lateral lobes narrow and posterior margin triangle-like (Fig. 3B); [3] ventral plate concave with a dorsal elevation in the middle extended to the virga; [4] virga basally with two slight tips.

###### Description.

*Male* (holotype). Body length including wings 7.55 mm; pronotum length 1.38 mm, pronotum width 1.64 mm; tegmina length 2.91 mm; hind wings length 5.84 mm; interocular distance 0.37 mm. (n=1) (Fig. 3A).

Head. Interocular distance more than half the eye width. Median ocellus fully developed. Internal margins of compound eyes convergent dorsally. Slight yellowish-white spot in the superior eyes corner. Gena below compound eye and antennae insertion black and below eye slightly yellowish. Maxillary palp black, distally slightly yellowish white, five segments with second reduced, fifth with strong setaes. Labial palp black.

Antennae thick and mainly black. Number of antennal segments 10. Scape wider than pedicel. Pedicel as long as 1^st^ flagellomere. Slight white distal spot on scape. White dorsal spot on pedicel. White dorsal spot on flagellomeres 1, 2 and 3. White dorsodistal spot on flagellomere 4. Flagellomeres 5, 6 and 7 black. Flagellomere 8 black with distal half completely white.

Thorax. Pronotum black with an anterior slender white line and an almost imperceptible yellowish at anterior corners. Tegmina black. Hind wing with white, transverse groove. Procoxa black. Protochanter black. Profemora black with a yellowish serrated distal inner lobe. Protibiae black with three distal spines. Mesocoxa black. Mesotrochanter black. Mesofemora black. Mesotibiae black distally brownish. Metafemora black; Semi-lunar process, metatibia and metatarsi brown.

Abdomen. Tergum 9 with a distal notch. Tergum 10 slightly concave, strongly sclerotized with erect vertical setae at edges (Fig. 3E). Cerci unsegmented, black. Brachium brownish, dorsolateral flat and wide with an inner protrusion. Brachium spine present. Epiproct lateral lobes narrow, covering base of cerci but not covered by tergum. Medial epiproct membranous, narrow and triangle-like (Fig. 3G). Uncus reduced without distal hook. Subgenital plate distally narrowed, constricted before end with conspicuous long and curved bristles (Fig. 3F).

Basal plate heavily sclerotized, very short and widened basally. Cingulum distally serrated without apodemes. Lateral valves pointed and serrated. Virga thick, distally rounded and serrated, basally with two slight tips. Ventral plate concave with a dorsal elevation in middle extended to virga (Fig. 3B).

*Variations*. Body length including wings 8.9 mm, excluding wings 7.2 mm; pronotum length 1.6 mm, pronotum width 1.8 mm; tegmina length 3.3 mm; hind wings length 6.3 mm; interocular distance 0.40 mm. (n=1). Antennae Scape black.

*Female*. Body similar to male except for abdominal sexual structures. Subgenital plate obtuse (Fig. 3C).

*Female variation*. body length including wings 8.5 mm, excluding wings 7.4 mm; pronotum length 1.4 mm, pronotum width 1.9 mm; tegmina length 3.5 mm; hind wings length 6.7 mm; interocular distance 0.43 mm. (n=1). Antennae: scape black, white dorsodistal spot on pedicel. White dorsal small spot on flagellomeres 1, 2 and 3.

###### Etymology.

The specific epithet is derived from the name of the type locality, Parque Nacional Natural Cueva de los Guácharos.

###### Distribution.

This species is currently known from the type locality.

###### Sympatric species.

This species was found in one of the malaise samples with the species *Ripipteryx diegoi* and *Ripipteryx ecuadoriensis*, which are believed to occur sympatrically.

###### Remarks.

*Ripipteryx guacharoensis* sp. n. is assigned to the Marginipennis group based on the characters of the phallic complex, such as the very short and broad basal plate, the cingulum without apodemes, the presence of lateral valves, and the thickened virga (Fig. 3B).

The new species is similar to *Ripipteryx femorata* in that both share a serrated distal inner lobe on the profemora, the shape of the male brachium in lateral view, the uncus reduced without distal hook and similar phallic complex (see Günther 1969). Nevertheless, it differs in the form of the ventral plate, which is concave in *Ripipteryx guacharoensis* but is straight in *Ripipteryx femorata*. The basal shape of the virga presents two slight tips in *Ripipteryx guacharoensis* while in *Ripipteryx femorata* it presents two strong and elongate tips (see Günther 1969). The most significant character separating both species is the posterior margin of the epiproct, which is triangular in *Ripipteryx guacharoensis* (Fig. 3G) but parabolic in *Ripipteryx femorata* (see Günther 1969).

According with Günther (1969) *Ripipteryx femorata* is closely related to *Ripipteryx vicina* and *Ripipteryx difformipes*. *Ripipteryx guacharoensis* shares with these three species the form of the subgenital plate that in males is distally constricted with conspicuous long and curved bristles, supporting a probable relationship.

#### Crassicornis Group *sensu* Heads, 2010

##### Ripipteryx gorgonaensis

Taxon classificationAnimaliaOrthopteraRipipterygidae

Baena-Bejarano & Heads sp. n.

http://zoobank.org/250F0723-AD54-4DCE-965F-71C5EA962171

Fig. 4

###### Holotype.

♂ (no. IAvH-E 113896), Colombia, Cauca, PNN Gorgona, Alto el Mirador, 2°58'N, 78°11'W, 180 m, Malaise, 6-20.ix.2000, Col. H. Torres. Specimen dried and pinned; deposited at Instituto Alexander von Humboldt, Villa de Leyva.

###### Paratypes.

5 specimens from same locality as holotype: 1) ♂ (no. IAvH-E 113898), 08–30.xi.2000, specimen dried and pinned; 2) ♂ (no. IAvH-E 113901), 01-04.iii.2000, Col. M. Sharkey, specimen dried and pinned; 3) ♂ (no. IAvH-E 113908), 18.i.2001, specimen dried and pinned; 4) ♂ (no. IAvH-E 113899), 30.x-18.xii.2000, specimen dried and pinned; 5) ♂ (no. IAvH-E 143179), 18.xii.2000-03.i.2001, specimen preserved in alcohol. Specimens deposited at same institution as holotype. 6) ♂ (no. GOR 3728-1), Colombia, Cauca, PNN Gorgona, Sendero cerro Trinidad, 2°58'22"N, 78°10'43"W, 90 m, Captura directa (manual), 21.x.2010, Col. F. Sarria. Specimen dried and pinned; deposited at Colección de insectos del PNN Gorgona - Museo de Entomología de la Universidad del Valle (MUSENUV), Cali.

###### Diagnosis.

The new species is almost cryptically similar to *Ripipteryx atra* Serville, 1838 sharing with it the coloration of the antennae (white spots on flagellomeres 1 and 4–7). However, it is readily separated from the latter species by [1] flagellomeres 1 and 2 free (not fused as in *Ripipteryx atra*); and [2] lateral lobes of epiproct with shallow lateral invagination.

###### Description.

**Figure 4.**
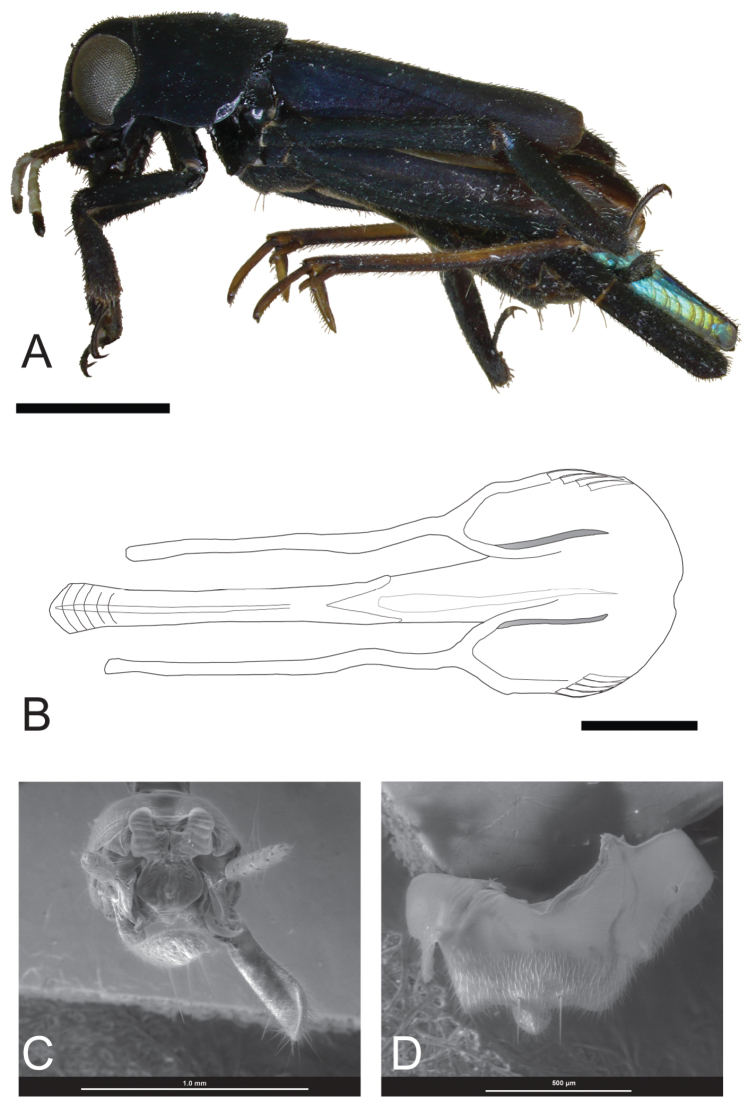
*Ripipteryx gorgonaensis* sp. n. male. **A** Holotype lateral habitus (IAvH-E 113896) (Scale bar 1.44 mm) **B** Dorsal view of phallic complex (IAvH-E 113899) (Scale bar 0.22 mm) **C** Scanning electron micrographs of dissected terminalia in frontal view (IAvH-E 143179) **D** Ventral view of subgenital plate.

*Male* (holotype). Body length including wings 6.80 mm, excluding wings 5.72 mm; pronotum length 1.34 mm, pronotum width 1.55 mm, tegmina length 2.84 mm, hind wings length 5.25 mm, interocular distance 0.41 mm (n=1) (Fig. 4A).

Head. Interocular distance more than half the eye width. Median ocellus fully developed. Patch that circumscribes the anterodorsal margin of compound eyes absent. Internal margins of compound eyes convergent dorsally. Patch of setae at posteroventral border of eye present. Maxillary palp black. Four maxillary palps. Labial palp black.

Antennae black and filiform. Number of antennae segments 10. Scape wider than pedicel. Pedicel shorter than 1^st^flagellomere. Flagellomere 2 shorter than 1. White spots on scape and pedicel absent. White dorsal spot on flagellomere 1 present. White dorsal spot on flagellomere 2 and 3 absent. White dorsal spot on flagellomere 4–7 present. White dorsal spot on flagellomere 8 absent. Color of gena below compound eye and antennae insertion black.

Thorax. Pronotum, mesonotum and tegmen black. White transversal groove on hind wings present. Procoxa black. Profemora black with a distal white spot. Protibiae black. Mesocoxa black. Ventral Mesotrochanter black. Mesofemora black. Mesotibiae black. Metafemora black. Semi-lunar process brown. Metatarsi brown.

Abdomen. Cerci unsegmented, black, spots absent. Brachium black, in lateral view with parallel sides. Brachium spine present. Subgenital plate with medial ridge (Fig. 4D). Lateral lobes of epiproct narrow with shallow lateral invagination, not covered by tergum. Medial epiproct tongue-like with a middle lobe (Fig. 4C). Uncus not embedded in brachium basis, 1-hooked.

Basal plate heavily sclerotized, long and narrow; strongly widened distally. Virga very slender not extended beyond cingulum. Cingulum well-sclerotized, but discontinuous; apodemes of cingulum elongate, at base 2-hooked (Fig. 4B).

*Variations*. Body length including wings 6.56–7.74 mm, excluding wings 5.11–7.48 mm; pronotum length 1.24–1.43 mm, pronotum width 1.45–1.66 mm; tegmina length 2.65–3.31 mm; hind wings length 4.97–5.77 mm; interocular distance 0.40–0.44 mm. (n=7).

*Female* unknown.

###### Etymology.

The specific epithet is derived from the name of the type locality.

###### Distribution.

This species is currently known from the type locality.

###### Sympatric species.

*Ripipteryx gorgonaensis* was found in sympatry with the species *Ripipteryx atra* and *Ripipteryx nodicornis*.

###### Remarks.

The terminalia and the subgenital plate of the new species resemble those of *Ripipteryx atra*, *Ripipteryx laticornis* Günther, 1963 and *Ripipteryx antennata* Hebard, 1924 suggesting placement in the Crassicornis Group. It shares the presence of numerous sharp spiculae on the cingulum with *Ripipteryx antennata* and *Ripipteryx atra* and the antennal color pattern with *Ripipteryx atra*. However, *Ripipteryx gorgonaensis* differs from the former species by the absence of modifications of the antennae. In other members of the Crassicornis Group, certain antennomeres are fused (e.g. in *Ripipteryx atra*) or otherwise modified (e.g. flattened and wide in *Ripipteryx laticornis* and *Ripipteryx antennata*); this is not the case in *Ripipteryx gorgonaensis*. The latter is easily distinguished from other species of the Crassicornis and Forceps groups by the form of the terminalia (Figs 4C, D).

A number of soft-bodied mites were found between the metanota and abdomina of some individuals. These are presumed to be ectoparasitic though further research is needed to clarify their biology and interaction with *Ripipteryx gorgonaensis* (O. Combita pers. comm.).

## Discussion

Five species groups had been proposed in the genus *Ripipteryx* which are largely defined by the morphology of the male terminalia and the phallic complex (Günther 1969; Heads 2010). Of the species described herein, *Ripipteryx diegoi* sp. n. and *Ripipteryx guacharoensis* sp. n. can be confidently assigned to the Forceps and Marginipennis species groups respectively based on coloration, body size and the morphology of the male terminalia and internal genitalia. In contrast, the species group placement of *Ripipteryx gorgonaensis* is problematic due to the presence of characters found in both the Crassicornis and Forceps groups such as modified subgenital plate and brachium, distal half of phallic complex weakly sclerotized, long apodemes of the cingulum, virga long and slender. *Ripipteryx gorgonaensis* was assigned to the Crassicornis group because it shares several characters of the terminalia with the species *Ripipteryx atra*, *Ripipteryx antennata* and *Ripipteryx laticornis* and possesses spines on the cingulum like other species in the group. However, it lacks antennal modifications (a diagnostic character of the Crassicornis group) with the antennae more similar to those of Forceps group species. In briefly reviewing Günther’s (1969) species group classification, Heads (2010) noted that the monophyly of some of the groups is questionable. Preliminary morphological phylogenetic analysis of the genus (Baena-Bejarano, unpublished) suggest that this is indeed the case, but more morphological and molecular data and a comprehensive phylogenetic treatment are required before a refined classification can be presented.

## Supplementary Material

XML Treatment for Ripipteryx diegoi

XML Treatment for Ripipteryx guacharoensis

XML Treatment for Ripipteryx gorgonaensis
